# Treatment of Anasarca

**Published:** 1881-06

**Authors:** 


### Selections.

---

### TREATMENT OF ANASARCA.

In the treatment of general anasarca in Bright's disease, the necessity of exciting the skin to action is universally recognized. By means of the perspiration, much fluid can be removed from the body and the œdematous condition of the patient relieved. In many cases where no danger exists of the occurrence of œdema of the lungs, pilocarpine may be used to produce this effect. In some cases it is contraindicated by organic or valvular disease of the heart. In these cases the following method is employed in the hospital: The patient is sponged off with alcohol, is then wrapped in a wet sheet, over which several blankets are placed. In the course of an hour the diaphoresis is usually profuse. The use of alcohol before the pack is recent, and has proven successful. It is supposed to act directly upon the sweat glands as a stimulant, and certainly increases the amount of the sweating produced by the pack. When these means do not produce sufficient diaphoresis, the fluid extract of jaborandi in dose of one drachm is given just before the pack is applied, and as it may produce nausea if given by the stomach, a preferable method of administration is by enema, in which case the dose may be increased to one and one-half drachms. In one case at present in the wards this method is daily pursued with good results. The anasarca is rapidly decreasing. When diaphoresis by the hot-air bath is attempted, the use of alcohol is found to be of equal service, and in cases of uræmic convulsions it has certainly hastened the excretion through the skin.—*Bellevue Hospital.*

